# Wip1 suppresses ovarian cancer metastasis through the ATM/AKT/Snail mediated signaling

**DOI:** 10.18632/oncotarget.8833

**Published:** 2016-04-18

**Authors:** Sheng Yin, Pan Wang, Lina Yang, Yang Liu, Yan Wang, Mingming Liu, Zihao Qi, Jiao Meng, Ting-Yan Shi, Gong Yang, Rongyu Zang

**Affiliations:** ^1^ Department of Gynecological Oncology, Fudan University Shanghai Cancer Center, Shanghai Medical College, Fudan University, Shanghai 200032, China; ^2^ Cancer Institute, Fudan University Shanghai Cancer Center, Shanghai Medical College, Fudan University, Shanghai 200032, China; ^3^ Ovarian Cancer Program, Division of Gynecologic Oncology, Department of Gynecology and Obstetrics, Zhongshan Hospital, Fudan University, Shanghai 200032, China; ^4^ Department of Gynecology and Obstetrics, The Fifth People's Hospital of Shanghai Fudan University, Shanghai 200240, China; ^5^ Central Laboratory, The Fifth People's Hospital of Shanghai Fudan University, Shanghai 200240, China

**Keywords:** Wip1, metastasis, ovarian cancer, EMT

## Abstract

Inactivation of p53 greatly contributes to serous ovarian cancer, while the role of the wild-type p53 induced phosphatase 1 (Wip1) is quite unclear. In this study, by silencing or overexpression of Wip1, we found that Wip1 suppressed ovarian cancer cell invasion, migration, epithelial to mesenchymal transition (EMT), and ovarian cancer metastasis in xenograft animal models. Mechanistic studies showed that Wip1 may block ovarian cancer metastasis through inhibition of Snail and p-Akt expression because silencing or overexpression of Wip1 either upregulated or downregulated the expression of Snail and p-Akt (Ser 473), while further knockdown of Snail by shRNA or inhibition of p-Akt by a chemical compound attenuated cell invasion, migration and EMT in Wip1 silencing cells. We also found that the phosphorylation of Akt at Ser 473 might be mediated through p-ATM (Ser 1981). Thus, Wip1 may suppress ovarian cancer metastasis through negative regulation of p-ATM, p-Akt, and Snail, which was also evidenced in the limited clinical specimens. Therefore, our data may provide a novel therapeutic indication for serous ovarian cancer based on the uncovered mechanism associated with the precise function of Wip1 independent of p53.

## INTRODUCTION

Ovarian cancer is the eighth most common cancer in women and the leading cause of death from gynecological malignancies in developed countries [1]. According to the American Cancer Society (Atlanta, GA), 21,980 new ovarian cancer cases were diagnosed in 2014, and the 5-year survival was around 44% [2]. Approximately 70% of ovarian cancer patients are diagnosed at the stage of FIGO III/IV with widespread cancer cells beyond ovaries. Despite surgical and chemotherapeutic improvements, most patients may eventually relapse and have poor prognosis after primary treatment. Numerous studies have demonstrated that EMT is associated with the motility or invasion of ovarian carcinoma. However, the detailed mechanism of EMT in ovarian cancer has yet to be defined.

Wip1, also called as *PPM1D* (protein phosphatase, Mg2+/Mn2+ dependent 1D), was first identified by Michele Fiscella and his colleges in 1997 [3]. Wip1 plays its role through dephosphorylating the downstream proteins including p38, p53, ATM, Chk2, and γ-H2AX that are involved in DNA damage response (DDR). Wip1 inactivates these molecules and turns back the cells to a homeostasis state when a process of DDR is completed [3–6]. Besides, Wip1 has recently been reported to be involved in regulation of cellular metastasis. Zhao et al. [7, 8] reported that Wip1 might negatively regulate neutrophil migration through regulating p38 MAPK activity. Li et al. [9] found that Wip1 knockout enhanced the migration of bone marrow mesenchymal stem cells. Paradoxically, several other studies suggest that Wip1 may enhance migration or invasion in some carcinomas [10, 11]. Although p53 is high mutated in serous ovarian cancer, the function of Wip1 in ovarian cancer metastasis is unclear.

In the present study, we investigated the biological function of Wip1 in ovarian cancer via both *in vitro* and *in vivo* assays. Our results suggest that Wip1 inhibits ovarian cancer metastasis through the ATM/Akt/Snail mediated signaling.

## RESULTS

### Knockdown of Wip1 promotes cell invasion and migration

**Figure 1:**
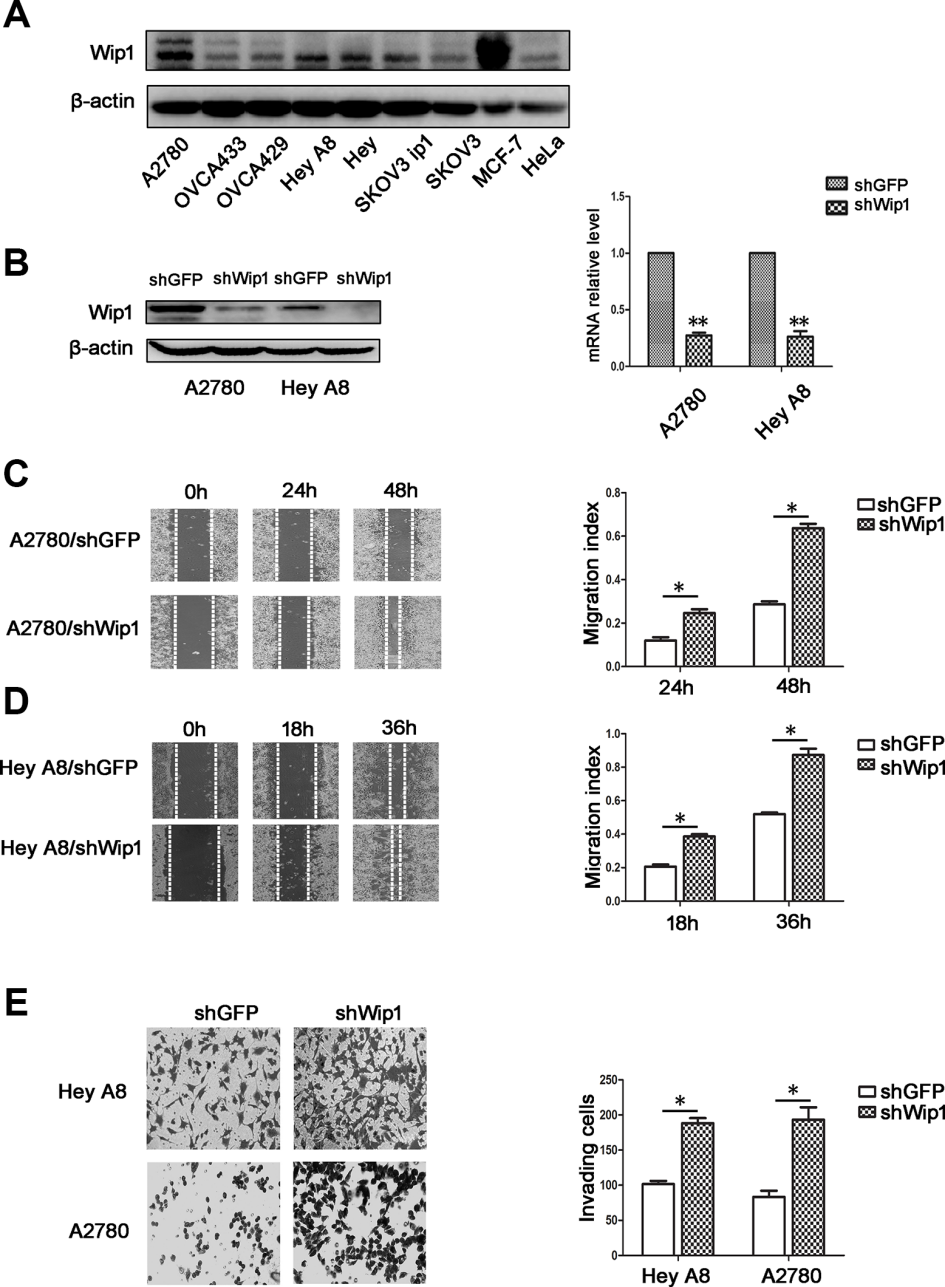
Knockdown of Wip1 promotes cell invasion and migration (**A**) Expression of Wip1 in A2780, OVCA433, OVCA429, Hey A8, Hey, SKOV3 ip1, SKOV3, MCF7 (a positive control) and Hela (a negative control) cell lines. (**B**) Efficiency of Wip1 shRNA detected by Western blotting and RT-PCR. **p < 0.01. Error bars = 95% CIs. (**C, D**) Migration assay. Wounds were imaged at 0 h, 24 h, and 48 h (A2780) and at 0 h, 18 h, and 36 h (HeyA8). *p < 0.05. Error bars = 95% CIs. (**E**) Invasion was detected by Trans-well experiments. Image was acquired at 24 h. *p < 0.05. Error bars = 95% CIs.

To examine the expression of Wip1, serous ovarian cancer cell lines A2780, Hey, HeyA8, SKOV3, SKOV3 ip1, OVCA433 and the clear cell carcinoma cell line OVCA429 were cultured and subjected to Western blotting analysis. MCF7 and HeLa cell lines were chosen as the positive and negative controls, respectively. According to Figure 1A, A2780 and HeyA8 cells were chosen for Wip1 knockdown experiments. Reconstructed plasmid pLKO.1-shWip1 and the control pLKO.1-shGFP were used to establish A2780/shWip1and HeyA8/shWip1 cell lines and the corresponding control cell lines A2780/shGFP and Hey A8/shGFP, which were verified by Western blotting and real time-PCR (Figure 1B). We found that knockdown of Wip1 increased cell migration (Figure 1C and Figure 1D) and invasion (Figure 1E), indicating that Wip1 may suppress cell invasion and migration in serous ovarian cancer cells.

### Wip1 suppresses EMT

**Figure 2:**
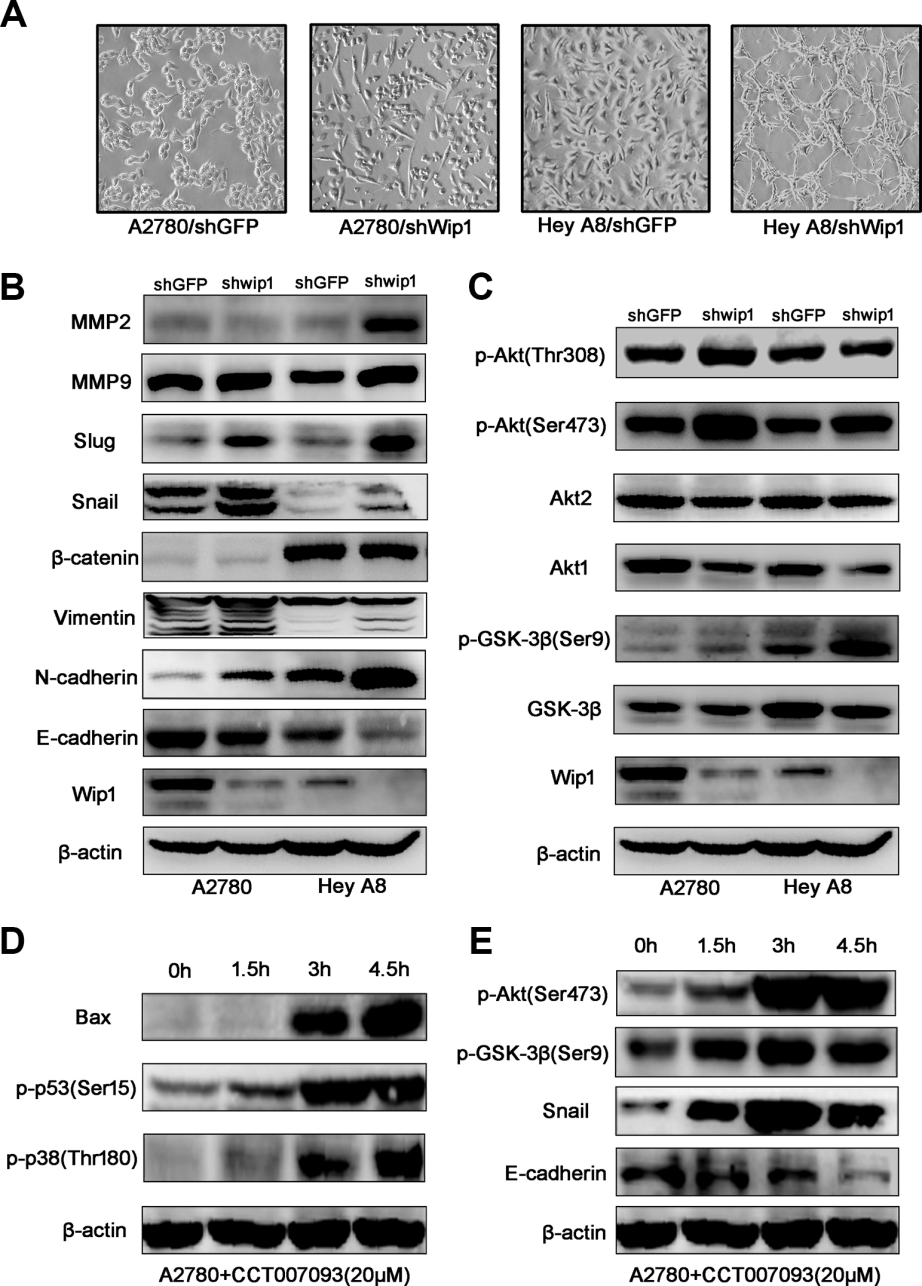
Wip1 knockdown activates EMT and the Akt/GSK-3β signaling (**A**) Morpgological change in cells expressing Wip1 shRNA. (**B**) Knockdown of Wip1 reduces E-cadherin, but increases N-cadherin, snail, slug, vimentin, MMP9, MMP2, but did not influence expression of β-catenin. (**C**) Wip1 knockdown promotes the phospharylation of GSK-3β (Ser9) and Akt (Ser473). (**D, E**) Treatment with CCT007093 activated the Akt/GSK-3β signaling pathway and EMT.

As shown in Figure 2A, cells expressing Wip1 shRNA displayed a thinner and longer morphology than did control cells expressing shGFP. To illustrate the potential mechanism, MMPs and EMT-related molecules were examined by using Western blotting. As shown in Figure 2B, knockdown of Wip1 decreased the expression of E-cadherin, while remarkably increased the expression of N-cadherin, snail and slug. The expression of vimentin, MMP9, MMP2 was slightly increased, but no changes of β-catenin were conceived. Since Wip1 is a serine-threonine protein phosphatase, we speculated that a serine/threonine protein kinase might be closely regulated by Wip1 through protein dephosphorylation.

Akt, a serine-threonine protein kinase, plays a central role in a variety of oncogenic processes. It is well known that the Akt activation associated with tumor metastasis is mediated through the phosphorylation at Ser473 [12]. Previous reports also suggest that the activation of Akt may induce the phosphorylation of GSK-3β to suppress the GSK-3β/snail/E-cadherin signaling [12–14]. Therefore, we tested the alterations of these molecules. As shown in Figure 2C, knockdown of Wip1 decreased the expression of Akt1 and GSK-3β, but increased the phosphorylation of GSK-3β at Ser9 and Akt at Ser473. However, no alterations of Akt2 and p-Akt (Thr308) were observed. To validate the above results, CCT007093 (10 μM), a specific inhibitor of Wip1 [15], was used to perform a time course inhibition assay. As shown in Figure 2D, treatment of cells with CCT007093 activated p38 and p53, and enhanced the expression of pro-apoptotic protein Bax, which is consistent with other reports [16, 17]. Meanwhile, CCT007093 treatment decreased E-cadherin and increased pGSK-3β^Ser9^ and pAkt^Ser473^ in a time-dependent manner (Figure 2E). These results revealed that the inhibition of Wip1 activity by using the specific inhibitor yielded a similar effect to shRNA transfection. Collectively, these data suggest that inhibition of Wip1 may activate the Akt/GSK-3β/snail signaling.

### Wip1 overexpression decreases cellular migration and invasion ability

**Figure 3:**
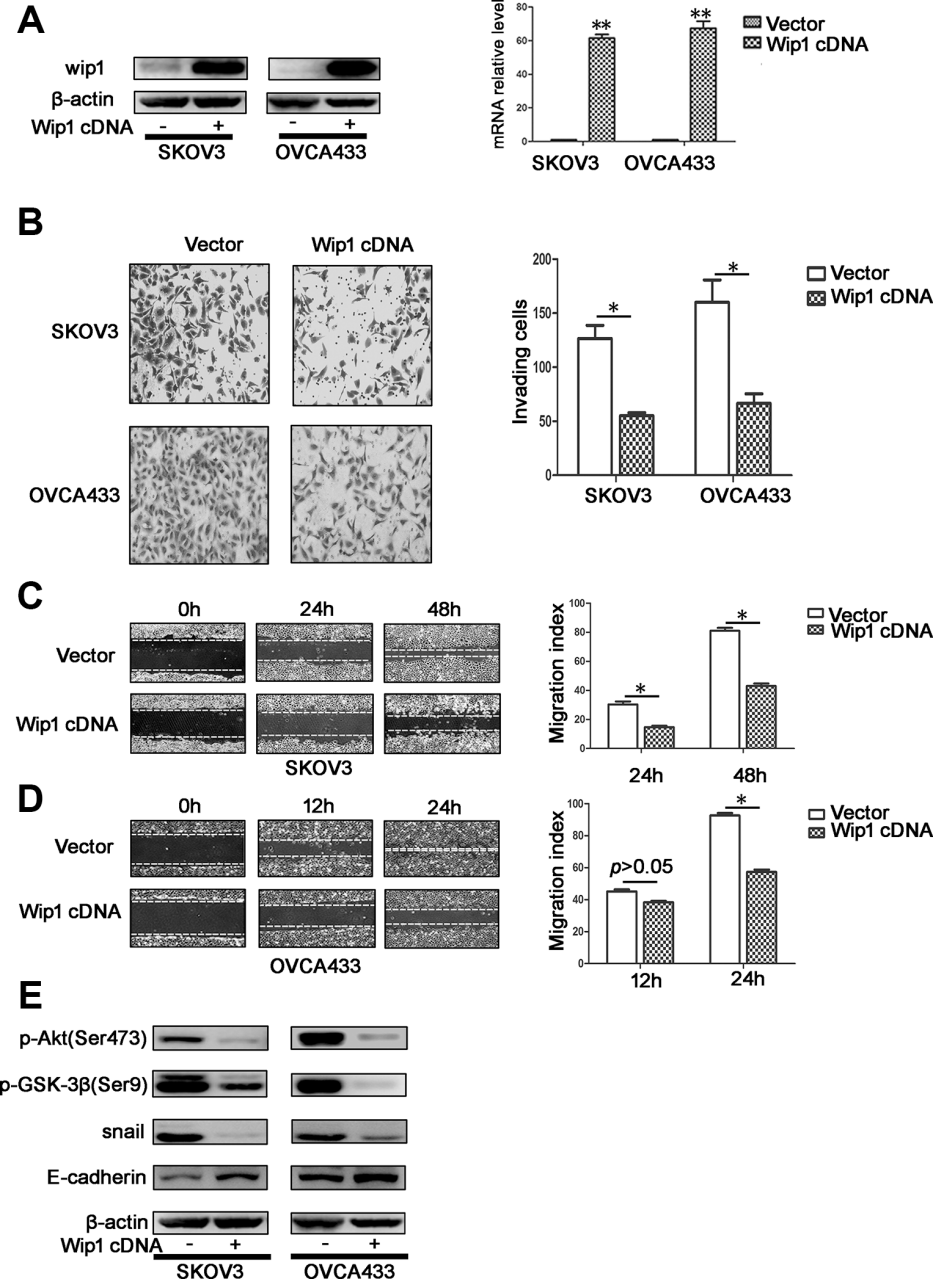
Overexpression of Wip1 decreases cellular migration and invasion *in vitro* (**A**) Overexpession of Wip1 detected by Western blotting and real time-PCT in SKOV3 and OVCA433 cell lines. **p < 0.01. Error bars = 95% CIs. (**B**) Cellular invasion was detected by trans-well experiment. The invaded cells were imaged at 24 h. *p < 0.05. Error bars = 95% CIs. (**C, D**) Cellular migration was detected by Scratch assay. The wounds were imaged at 0 h, 24 h, 48 h (SKOV3) and at 0 h, 12 h, 24 h (OVCA433). *p < 0.05. Error bars = 95% CIs. (**E**) Overexpression of Wip1 suppressed Akt/GSK-3β signaling and EMT.

As silencing of Wip1 promoted metastasis in serous ovarian cancer cells, we examined whether overexpression of Wip1 could suppress cellular motility. Thus, the pCDH-*PPM1D* plasmid was generated and transfected into SKOV3 and OVCA433 cells. Compared with in control cells, the expression of Wip1 was dramatically increased in SKOV3/Wip1 cDNA and OVCA433/Wip1 cDNA cells, which was assessed by Western blotting and real time-PCR (Figure 3A). Remarkably, overexpression of Wip1 decreased invasion and migration *in vitro* (Figure 3B–3D) by upregulation of E-cadherin and down regulation of snail, pAkt^Ser473^, and pGSK-3β^Ser9^ (Figure 3E).

### Wip1 negatively regulates tumor metastasis

**Figure 4:**
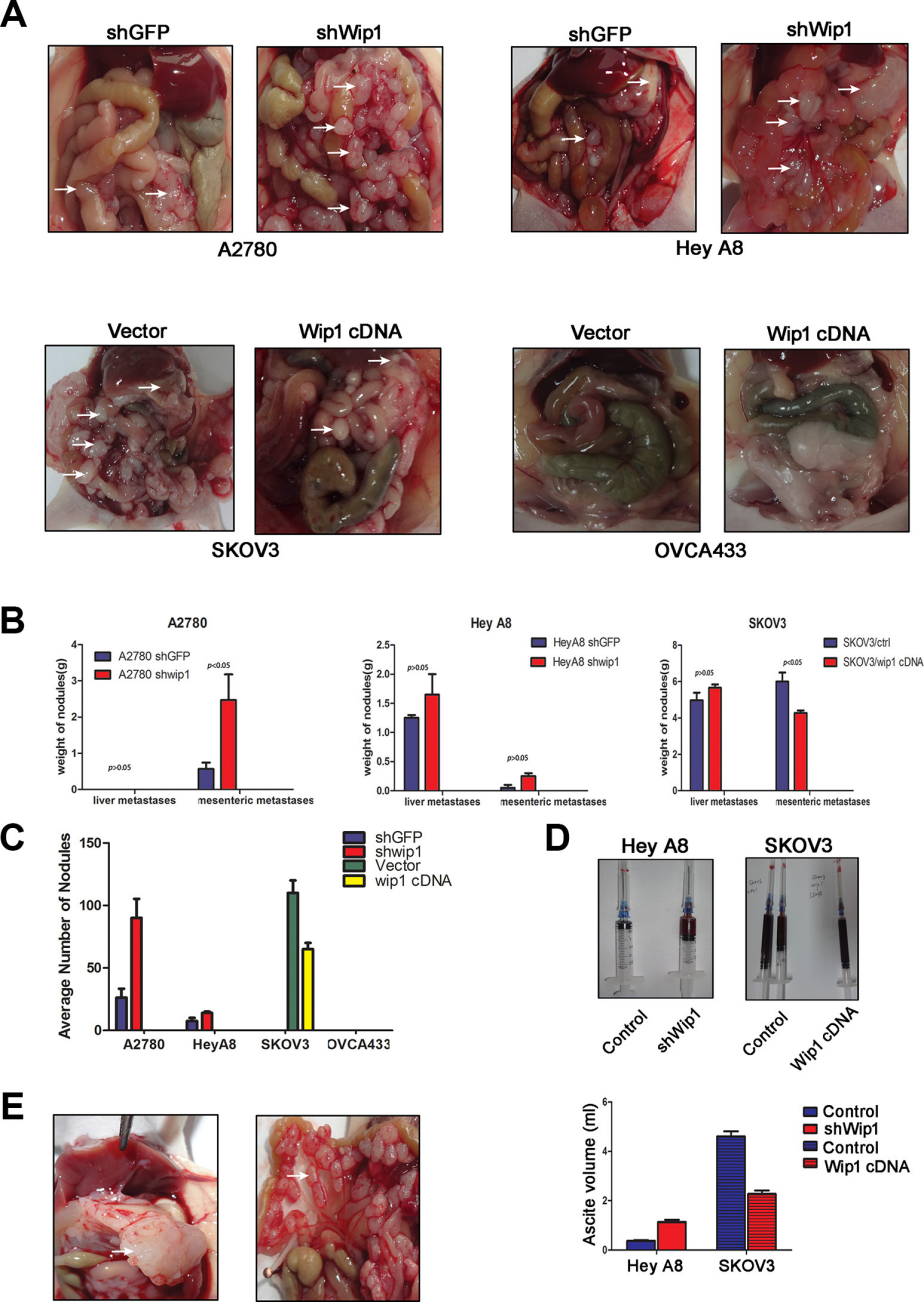
Wip1 suppresses tumor metastasis *in vivo* (**A**) In intraperitoneal models, knockdown of Wip1 promotes formation of metastatic nodules in small bowels and mesenteric surface and liver (A2780 and Hey A8). Overexpression of Wip1 in SKOV3 decreased mesenteric metastases. Injection of OVCA433 did not form tumors *in vivo*. White arrows referred to metastatic loci. (**B**) Weight of nodules. Error bars = 95% CIs. (**C**) Quantification of nodules. Error bars = 95% CIs. (**D**) Measurement of ascite volume. (**E**) Different metastatic patterns in our experiment: liver metastasis and mesenteric metastasis.

To test how Wip1 regulates tumor metastasis *in vivo*, animals were introperitoneally injected with A2780 shWip1, Hey A8 shWip1, SKOV3/Wip1 cDNA and OVCA433/Wip1 cDNA cells and the corresponding control cells, respectively. The mice were sacrificed before natural death, and the number of nodules and their weights were measured. As shown in Figure 4A, we found that the intraperitneal injection of A2780 cells only caused mesenteric metastases, but no liver metastases. Knockdown of Wip1 increased mesenteric metastases in A2780 cells, including both the weight of tumor nodules (Figure 4B) and average numbers (Figure 4C). Introperitoneal injection of Hey A8 or SKOV3 cells presented both mesenteric and liver metastases (Figure 4A). Wip1 knockdown in Hey A8 cells increased both liver tumor burden and mesenteric metastases although there was not statistical significance (Figure 4B and 4C). On the contrary, overexpression of Wip1 in SKOV3 cells decreased mesenteric metastases (*p* < 0.05), but did not show significant differences compared with liver metastases (Figure 4B and 4C). Both xenograft mice injected with OVCA433/Wip1 cDNA and control cells did not form any tumors *in vivo* (Figure 4A). Meanwhile, as shown in Figure 4D, knockdown of Wip1 in Hey A8 cells increased the volume of ascites, while overexpression of Wip1 in SKOV3 cells decreased the volume of ascites. Two different patterns of mesenteric metastases and liver metastases were shown in Figure 4E.

### Wip1 supresses cellular motility through the Akt/GSK-3β/snail signaling

**Figure 5:**
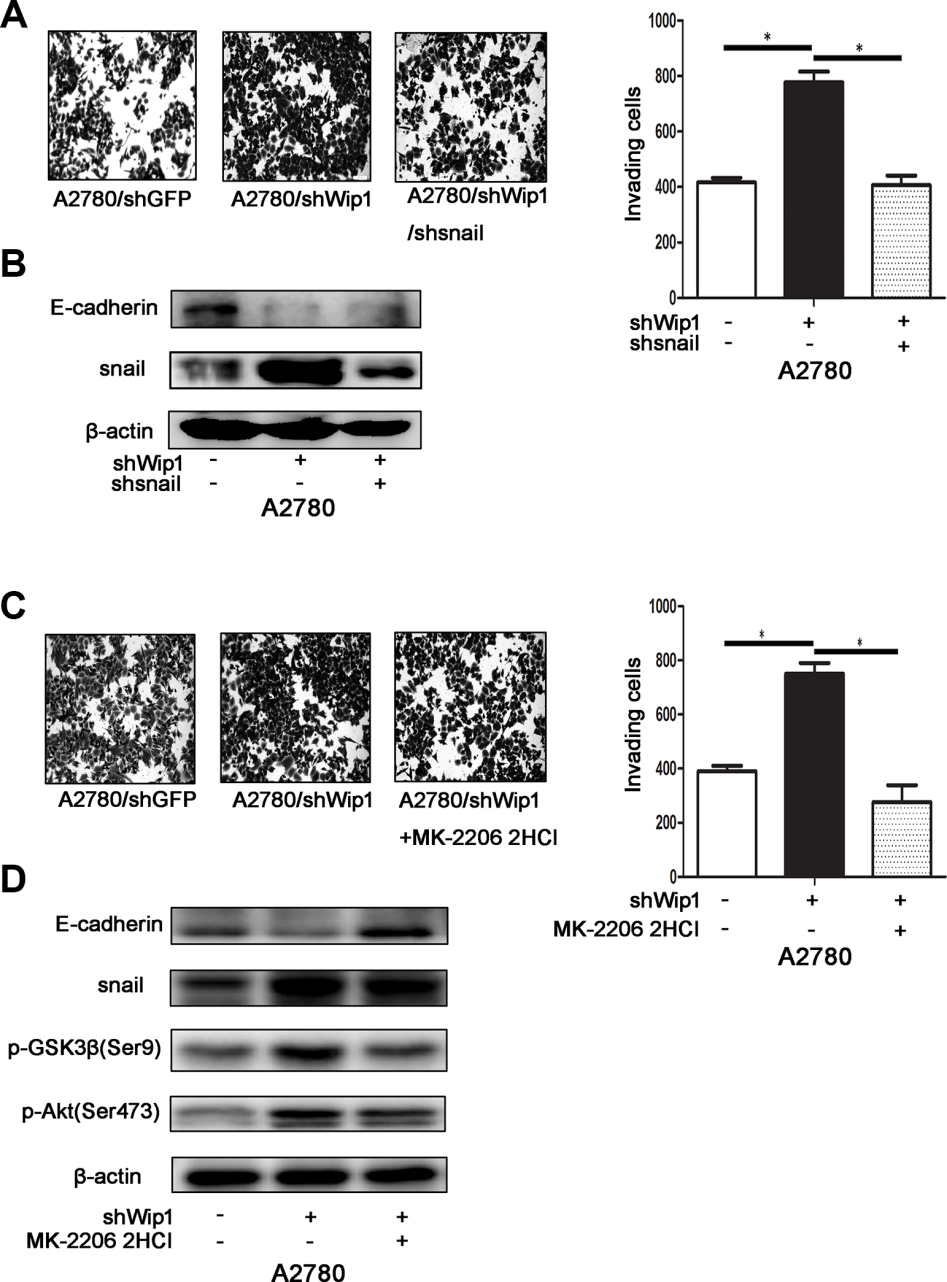
Wip1 suppresses cellular motility through Akt/GSK-3β/snail pathway signaling (**A**) Knockdown of snail in A2780/shWip1 neutralized the increased invading cells. Image was acquired at 36 h. *p < 0.05. Error bars = 95% CIs. (**B**) The decreased expression of E-cadherin Snail knockdown neutralized. (**C**) Cell invasion of A2780/shWip1 after MK-2206 2HCl treatment. Image was acquired at 36 h. *p < 0.05. Error bars = 95% CIs. (**D**) Detection of pGSK-3β^Ser9^, snail and E-cadherin in cells treated with MK-2206 2HCl.

The above data indicate that snail may be the major transcriptional factor during EMT process of ovarian carcinoma cells. To test whether snail truly contributes to the metastasis induced by Wip1 knockdown, we transiently transfected snail shRNA into the A2780/shWip1 cells. As shown in Figure 5A, knockdown of snail attenuated the cellular invasion of A2780/shWip1 cells. The expression level of E-cadherin was also increased in snail silencing cells compared with in control cells (Figure 5B). The above results suggest that the EMT transcriptional factor snail may be a direct downstream target of Wip1.

Similarly, to verify if the Akt/GSK-3β signaling essentailly promotes the snail mediated cell metastasis, we used the Akt inhibitor MK-2206 2HCl to inhibit the phosphorylation of Akt at Ser473 in A2780/shWip1 cells. As in Figure 5C, MK-2206 2HCl treatment also reduced the invasive ability of A2780/shWip1 cells. Both pGSK-3β^Ser9^ and snail were downregulated after MK-2206 2HCl treatment, while the expression level of E-cadherin was increased (Figure 5D).

### ATM links Wip1 and the Akt/GSK-3β/snail signaling

To analyze the physical interaction between Wip1 and Akt/GSK-3β, we performed co-immunoprecipitation experiment. However, the binding between Akt or GSK-3β with Wip1 was not found (Data not shown). We therefore speculate other proteins may mediate the activation of Akt/ GSK-3β.

**Figure 6:**
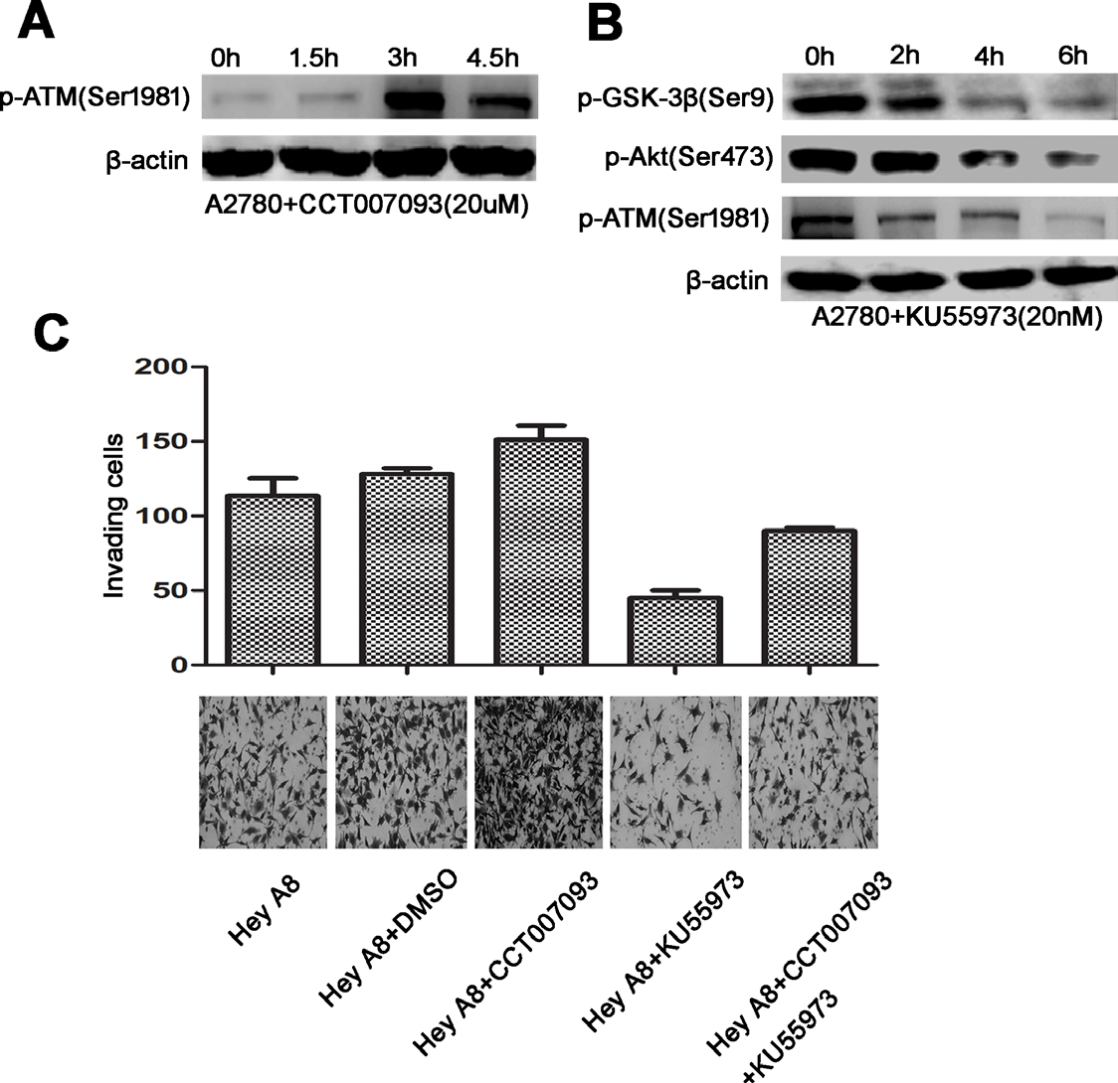
ATM links Wip1 and the Akt/GSK-3β/snail signaling (**A**) Inhibition of Wip1 by CCT007093 increased ATM activation in a time-dependent manner. (**B**) KU55973 suppressed the Akt/GSK-3β signaling. Cell pellets for Western blotting were collected at 0 h, 2 h, 4 h, and 6 h. (**C**) Invasion array for Hey A8: Inhibition of Wip1 by CCT007093 treatment increased the number of trans-membrane cells (*p* < 0.05). Error bars = 95% CIs. Inhibition of ATM by KU55973 suppressed cellular invasion (*p* < 0.05). Error bars = 95% CIs. Co-treatment of Hey A8 cells with CCT007093 and KU55973 neutralized the increased invading cells compared with Hey A8+CCT007093 (*p* < 0.05). Error bars = 95% CIs.

ATM, an important kinase that is activated through serine phosphorylation during DNA damage response, has been reported to be dephosphorylated by Wip1 at Ser1981 [5]. Furthermore, the PI3K domain of ATM could specifically regulate Akt (Ser473) phosphorylation [18]. To verify the potential role of ATM in the Akt/GSK-3β signaling, p-ATM was detected after cells were treated with CCT007093. As shown in Figure 6A, inhibition of Wip1 increased ATM activation in a time-dependent manner, which was consistent with a previous study [5]. We further used KU55933 [19], a specific ATM inhibitor, to evaluate the relationship between ATM and Akt. As shown in Figure 6B, the expression levels of p-Akt^Ser473^ and pGSK-3β^Ser9^ were markedly decreased in cells treated with KU55933, indicating that ATM may activate the Akt/GSK-3β signaling.

To further illustrate the impact of the Wip1/ATM/Akt signaling on cellular metastasis, we performed an invasion assay by using the high-invasive cell line Hey A8. As in Figure 6C, inhibition of Wip1 increased the number of trans-membrane cells, while inhibition of ATM suppressed the cellular invasion through rescuing the effect of Wip1 inhibition. These data suggest that the ATM/Akt mediated signaling may exert important functions on ovarian cancer metastasis in association with Wip1.

### Correlation of Wip1with p-ATM, p-AKT, snail and E-cadherin in ovarian cancer tissues

**Figure 7:**
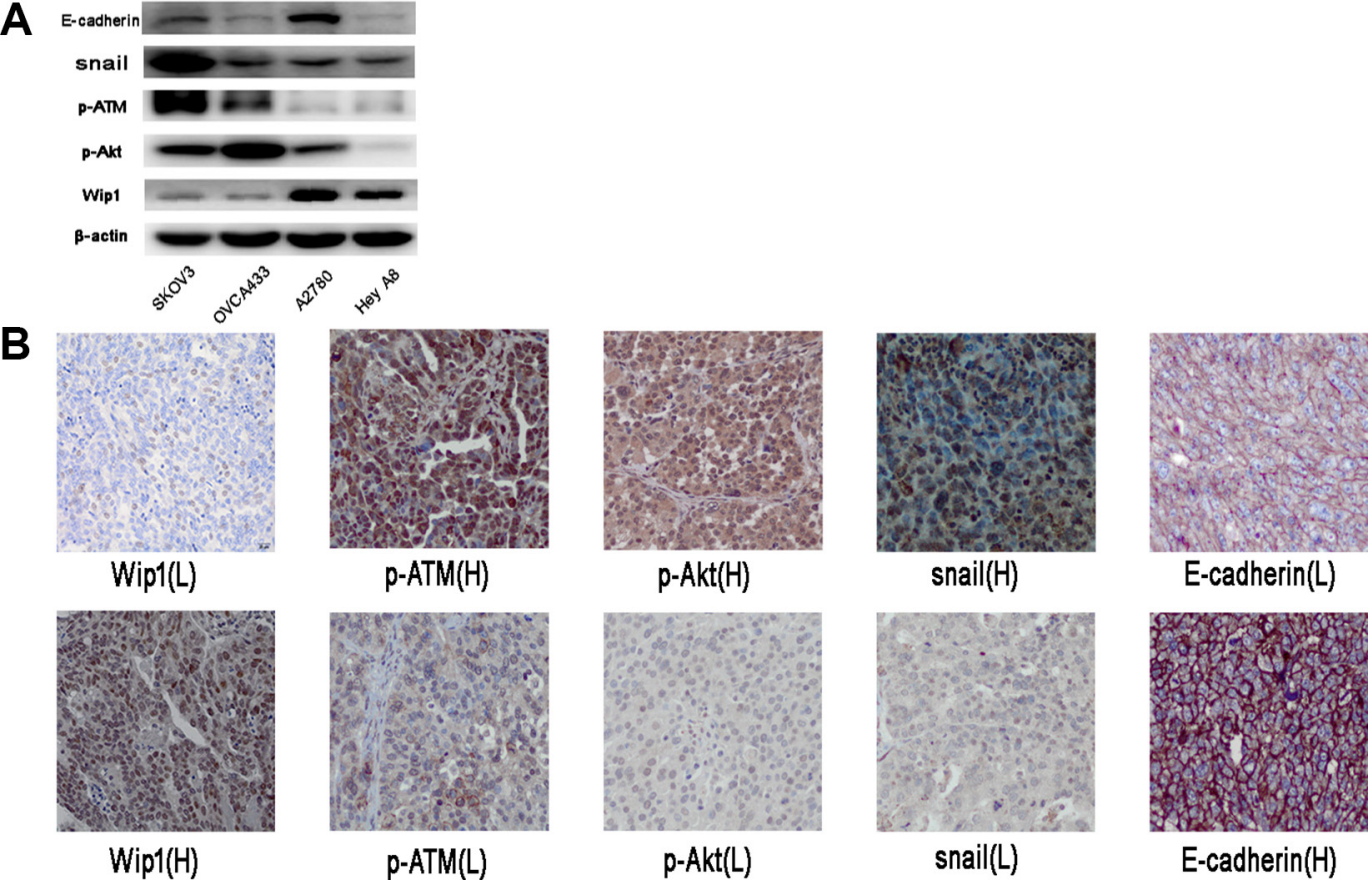
Correlation of Wip1with p-ATM, p-AKT, snail and E-cadherin in ovarian cancer tissues (**A**) Endogenous basal level of Wip1 and pAKT, SNAIL, p-ATM, E-cadherin in SKOV3, OVCA433, A2780 and Hey A8. (**B**) Representative images showing the level of Wip1, p-ATM, pAKT, snail and E-cadherin in the same tissue.

We next tested the endogenous basal level of Wip1 and pAKT, SNAIL, p-ATM, E-cadherin in the ovarian cancer cell lines. As shown in Figure 7A, Wip1 was correlated with the endogenous expression of p-ATM, pAKT, snail, but not E-cadherin To investigate the correlation of Wip1 with p-ATM, p-AKT, snail and E-cadherin in ovarian cancer tissues, we performed immunostaining using antibodies against Wip1, p-ATM, p-AKT, snail and E-cadherin in 53 high-grade serous ovarian cancer specimens. As shown In Table 1, the expression of Wip1 was negatively correlated with p-ATM, p-AKT, snail (*p* < 0.05), while positively correlated with E-cadherin expression, although the *p* value > 0.05. Representative images in the same tissue were shown in Figure 7B.

**Table 1:** Correlation of Wip1with p-ATM, p-AKT, snail and E-cadherin in ovarian cancer tissues

*N* = 53	p-ATM		p-AKT		snail		E-cadherin	
**Wip1**	Low	High	Low	High	Low	High	Low	High
Low	9	23	4	28	8	24	26	6
High	14	7	9	12	13	8	16	5
***P* value**	0.004		0.021		0.038		0.454	

## DISCUSSION

**Figure 8:**
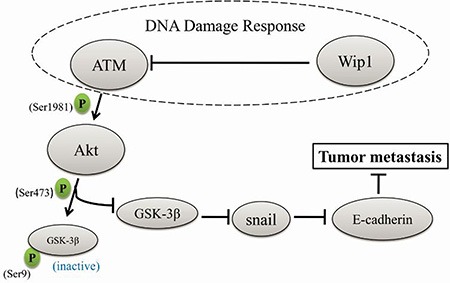
A schematic diagram of how Wip1 suppresses cellular metastasis through the ATM/Akt/Snail mediated signaling Both Wip1 and ATM are important regulators in DDR. Wip1 dephosphorylates ATM at Ser1981, and ATM activation phosphorylates Akt at Ser473. Akt activation can phosphorylate GSK-3β at Ser9, which lead to the degradation of GSK-3β, while GSK-3β attenuates snail expression and upregulates E-cadherin expression.

Our study show that knockdown of Wip1 facilitates an epithelial-mesenchymal transition through the activation of the Akt/GSK-3β signaling, leading to the enhanced invasion, migration and metastasis of serous ovarian cancer cells, while overexpression of Wip1 entirely reverses these effects. Since knockdown of snail and inhibition of the Akt/GSK-3β signaling inhibited the motility of cells with Wip1 knockdown, we propose that Akt, GSK-3β, and snail are downstream targets of Wip1, while ATM may link Wip1 with the Akt/GSK-3β/snail signaling (Figure 8).

Previous studies have shown that Wip1 regulates cellular metastasis in different cell types including neutraphil [7, 8], bone marrow mesenchymal stem cell [9], salivary adenoid cystic carcinoma [10], while we show here that Wip1 suppresses cellular metastasis in ovarian carcinoma. Despite the oncogenic role of Wip1 was reported in others carcinomas, we revealed an antitumor function of Wip1 in serous ovarian cancer cells. Since Wip1 overexpression in both p53 null (SKOV3) and wild type (OVCA433) cell lines suppressed the cellul motility in our results, we presume that Wip1 may also inhibit ovarian cancer metastasis in a p53-independent manner.

In advanced ovarian cancer patients, cancer cells always seed into the peritoneal cavity, we demonstrated that knockdown of Wip1 in both A2780 and Hey A8 cells increased the tumor nodules located at mesenteric or liver, while Wip1 overexpression in SKOV3 cells alleviated the tumor burden. The fact that the volume of ascites was affected by Wip1 knockdown or overexpression suggests a potential role of Wip1 in tumor microenvironment. These results may be explained by Zhao et al. [8] that neutrophils in Wip1-deficient mice produce more cytokines (IL-6, TNF-α, IL-1β, and so on) and granule proteins (elastase, lactoferrin, MPO, and MMP9) after LPS stimulation. Therefore, Wip1 overexpression may be a potential therapeutic choice to block the metastasis and recurrence of serous ovarian cancer, however, further study is needed to better understand the function of Wip1 in tumor microenvironment.

Taken together, our study suggest that Wip1 blocks cellular metastasis of serous ovarian cancer through the ATM/Akt/snail mediated signaling, which may be used to improve the efficiency of ovarian cancer treatment.

## MATERIALS AND METHODS

### Cell Lines and cell culture

Ovarian cancer lines A2780, OVCA429, Hey, HeyA8, SKOV3, SKOV3 ip1, OVCA433, breast cell line MCF7, cervical cancer cell line HeLa, and lentiviral packaging cell line 293T were obtained from the American Type Culture Collection (ATCC). 293T cell was cultured in DMEM media, and the other cells were cultured in RPMI 1640 medium supplemented with 10% (v/v) FBS and 1% penicillin and streptomycin at 37°C under a humidified condition with 95% air and 5% CO_2_.

### Plasmid constructs

DNA oligonucleotides used to generate shRNAs against the open reading frame of mRNA were 5′-CCCTTCTCGTGTTTGCTTAAA-3′ (for Wip1) and 5′-GACTACCGCTGCTCCATTCCA-3′ (for snail) to silence the expression of Wip1 and snail respectively. The shRNAs were cloned into pLKO.1 TRC Cloning Vector (Addgene) according to the manufacturer's protocol. The pLKO.1-shGFP was used as a control vector.

The pCMV6-AN-HA plasmid (Origene) containing full-length wild-type *PPM1D* cDNA was kindly gifted from Dr Nazneen Rahman [20]. The full-length wild-type *PPM1D* was amplified using the following: Forward: 5′-CTAGCTAGCATGGCGGGGCT GTACTCGCTGGGA-3′ and Reverse: 5′-ATAAGAATG CGGCCGC TCAGCAAACACAAACAGTTTTCCT-3′, the PCR product was digested with *Nhe1* and *Not1* and then cloned into the pCDH–CMV-MCS-EF1-PURO (Addgene), yielding pCDH-*PPM1D* construct. The pCDH empty vector was used as a control vector.

### Lentivirus infection and transient transfection with Plasmid DNA

Lentiviruses harboring shWip1 or Wip1 cDNA were produced by transfecting pLKO.1/shWip1 or pCDH-PPM1D into 293T cells. Serous ovarian cancer cells were infected twice for a total of 6 days (3 days for each infection) and the positive clones were selected with puromycin for 10–14 days to establish stable cell lines expressing shWip1 or Wip1 cDNA and the corresponding control vectors. The shRNA for snail was transiently transfected into cells by FuGENE HD (Roche) according to the manufacturer's protocol.

### Cell invasion and migration

Cell invasion was assessed using a 24-well inserts (BD Biosciences) with an 8-μm pores according to the manufacturer's instructions. Briefly, 5 × 10^4^ cells were seeded onto upper chamber which contained the Matrigel layer and allowed to invade at 37°C for 24 h toward a lower reservoir with 10% FBS. Cells were then fixed in 100% methanol for 30 minutes and stained with Crystal violet for 10 minutes. The invasive cells were counted as those passed through the membrane. All cells were counted at ×200 magnification under a microscope.

To examine cell migration, cells were incubated in 6-well plate over night to yield monolayer of 100% confluence for scratch assay. Scratched were made using a pipette tip and photographed immediately (time 0), and at 18/24 h and 36/48 h. The distance migrated by cells monolayer to close the scratch area during the time period was measured. Both the assays were repeated three times in duplicate. Migration index is defined as the distance migrated by shRNA or cDNA treated relative to the distance migrated by the control.

The snail shRNA (4 μg) was transiently transfected into A2780/shWip1 with FuGENE HD (12 μl) for 48 hours, then the cell culture medium was removed and cells were collected for the trans-well experiments.

### Real time fluorescence quantitative polymerase chain reaction

Total RNA was isolated with Trizol reagent (Invitrogen), and 1 μg RNA was reversely transcribed into cDNA using the Exscript RT-PCR kit (TaKaRa) according to the manufacturer's instructions. Primer pairs for cDNA amplification were as follows: 5′ -GCCAGAACTTCCCAAGGAAAG-3′ (forward) and 5′-GGTTCAGGTGACACCACAAATTC-3′ (reverse) for Wip1; 5′-GGCCTCCAAGGAGTAAGACC-3′ (forward) and 5′-CAAGGGGTCTACATGGCAAC-3′ (reverse) for GA PDH. All amplifications and detections were carried out in the Applied Biosystems Prism 7900 system (Applied Biosystems, Foster City, CA) using the ExScript Sybr green QPCR kit (TaKaRa, Japan) and the following program: 1 cycle of 15min at 95°C followed by 35 cycles of (10 sec at 95°C, 20 sec at 60°C). Statisticalanalyses were performed using the 2^−ΔΔCT^ relative quantification method. The assay was performed three times in triplicate.

### Western analysis

Cell lysates containing 30ug protein were analyzed. The following primary antibodies were used: antibodies to Wip1 (H300), p-p53 (Ser15), Bax, E-cadherin, N-cadherin, β-catenin, slug, snail, vimentin, Akt1, Akt2, p-Akt (Ser473), MMP9 and MMP2 were from Santa Cruz. Antibodies for β-actin, p-p38 (Thr180), p-Akt (Thr308) were from CST (Cell Signaling Technologies), GSK-3β, p-GSK-3β (Ser9) were from BD biosciences, p-ATM (Ser1981) was from Abcam. Immunoreactivity was conducted according to standard procedures.

### Inhibitor assay

CCT007093 (sigma-Aldrich, C9369), a Wip1 inhibitor, was prepared in DMSO at a stock concentration of 10 mM. A2780 was detached by trypsinization and washed with PBS twice. Same number of cells were plated in 6 wells dish for 24 h, and incubated with 20 μM CCT007093 for 0 h, 1.5 h, 3 h, and 4.5 h, then the samples were collected for Western blotting.

KU55933 (Selleck chem, S1092), an ATM inhibitor, was prepared in DMSO at a stock concentration of 50 μM. Same number of A2780 cells was incubated with 20 nM KU55933 for 0 h, 2 h, 4 h, and 6 h respectively, cell pellets were collected for Western blotting. For the invasion assay, same number of HeyA8 was plated into 24-well plate overnight and incubated with RPMI 1640 medium, RPMI 1640 + DMSO, RPMI 1640 + CCT007093 (20 μM), RPMI 1640 + KU55933 (20 nM), RPMI 1640 + CCT007093 (20 μM) + KU55933 (20 nM) respectively for 2 h, then those cells were collected for trans-well experiment.

MK-2206 2HCl (Selleck chem, S1078), an Akt inhibitor, was prepared in DMSO at a stock concentration of 10 mM. A2780/shWip1 was incubated with MK-2206 2HCl (10 nM) for 24 h, then cells were collected for invasion assay.

### Animal experiments

In intraperitoneal tumor model, four mice (Balb/c nude mice) were used for each cell line and each mouse received one injection of 1 × 10^7^cells. Mice were observed for the lethargy and abdominal enlargement every three days, and sacrificed before natural death occured. Tumor nodules and ascites were counted, weighed and measured.

### Imunohistochemical staining

Ovarian tissue samples from 53 high grade serous ovarian cancer patients who underwent primary debulking surgery at Fudan University Shanghai Cancer Center were included in our study. The expression of Wip1, p-ATM (Ser1981), p-AKT (Ser473), snail and E-cadherin was detected by immunohistochemical staining. The expression levels were judged based on the intensity of staning (IS) and the percentage of positive cells (PPC) as previously reported [21]. The final score (PPC×IS) was graded as “Low” for < 4 and “High” for ≥ 4.
